# Dissecting the Clinical Heterogeneity of Autism Spectrum Disorders through Defined Genotypes

**DOI:** 10.1371/journal.pone.0010887

**Published:** 2010-05-28

**Authors:** Hilgo Bruining, Leo de Sonneville, Hanna Swaab, Maretha de Jonge, Martien Kas, Herman van Engeland, Jacob Vorstman

**Affiliations:** 1 Department of Children and Adolescent Psychiatry, Rudolf Magnus Institute of Neuroscience, University Medical Centre Utrecht, Utrecht, The Netherlands; 2 Department of Clinical Child and Adolescent Studies, University of Leiden, Leiden, The Netherlands; 3 Department of Neuroscience and Pharmacology, Rudolf Magnus Institute of Neuroscience, University Medical Centre Utrecht, Utrecht, The Netherlands; Health Canada, Canada

## Abstract

**Background:**

The etiology of autism spectrum disorders (ASD) is largely determined by different genetic factors of variable impact. This genetic heterogeneity could be a factor to explain the clinical heterogeneity of autism spectrum disorders. Here, a first attempt is made to assess whether genetically more homogeneous ASD groups are associated with decreased phenotypic heterogeneity with respect to their autistic symptom profile.

**Methodology:**

The autistic phenotypes of ASD subjects with 22q11 deletion syndrome (22q11DS) and ASD subjects with Klinefelter Syndrome (KS) were statistically compared to the symptom profile of a large (genetically) heterogeneous ASD sample. Autism diagnostic interview-revised (ADI-R) variables were entered in different statistical analyses to assess differences in symptom homogeneity and the feasibility of discrimination of group-specific ASD-symptom profiles.

**Principal Findings:**

The results showed substantially higher symptom homogeneity in both the genetic disorder ASD groups in comparison to the heterogeneous ASD sample. In addition, a robust discrimination between 22q11-ASD and KS-ASD and idiopathic ASD phenotypes was feasible on the basis of a reduced number of autistic scales and symptoms. The lack of overlap in discriminating subscales and symptoms between KS-ASD and 22q11DS-ASD suggests that their autistic symptom profiles cluster around different points in the total diagnostic space of profiles present in the general ASD population.

**Conclusion:**

The findings of the current study indicate that the clinical heterogeneity of ASDs may be reduced when subgroups based on a specific genotype are extracted from the idiopathic ASD population. The current strategy involving the widely used ADI-R offers a relatively straightforward possibility for assessing genotype-phenotype ASD relationships. Reverse phenotype strategies are becoming more feasible, given the accumulating evidence for the existence of genetic variants of large effect in a substantial proportion of the ASD population.

## Introduction

Autism spectrum disorders (ASDs) delineate a group of behaviorally-defined disorders including autism, PDD-NOS, and Asperger syndrome.

Many efforts are being made to address the clinical heterogeneity of ASDs. At the same time, the diversity of genetic findings in the past decade indicate that ASDs should also be considered genetically heterogeneous [1], [2]. This raises the question to what extent the clinical heterogeneity can be explained by the underlying genetic heterogeneity of ASDs. In this study we will address this issue through the assessment of the homogeneity of the ASD phenotype in genetically more homogenous samples.

In addition to the growing number of genetic ASD susceptibility loci with small effect sizes, recent studies have described new “causative” genetic variants in ASDs that are assumed to have a large impact on ASDs [3], [4]. They are thought account for about 10–20% of ASD cases [1], [4], [5]. These risk variants are likely to show incomplete penetrance and imperfect segregation with disease as most variants have also been observed in non-autistic controls [5], [6]. Furthermore, several ASD variants have been shown to cause brain disorders other than ASD, including schizophrenia, mental retardation and epilepsy [1], [6]. This combination of incomplete penetrance and pleiotropic phenotypes could indicate that these loci cause a global disruption in brain development, making it more vulnerable to develop a range of different brain disorders. Efforts are required to distinguish distinct aspects of those brain disorders that are caused by these genetic variants with large effect, versus aspects that result from various other (environmental and/or genetic) hits.

A logical starting point would be to assess whether at all, ASD cases ascertained for a particular genetic variant display distinct autistic characteristics. This model can be considered probable when cases carrying the same genetic variant are found to share particular (combinations of) symptoms in higher frequencies than most cases in the idiopathic ASD population. The probability of genetic ASD subphenotypes can be illustrated by Rett syndrome (RTT). A more homogeneous profile of autistic symptoms together with non-autistic symptoms has led to the description of the RTT genetic subphenotype that is formally classified in the DSM-IV-TR as an ASD subtype. RTT is a progressive neurodevelopmental disorder that manifests in girls during early childhood [7], [8]. Mutations in *MECP2* gene are found in more than 95% of classic RTT cases [9], [10]. Patients with RTT appear to develop normally up to 6–18 months of age. Deceleration of head growth is often the presenting symptom. This is often accompanied by general growth retardation, weight loss, and a weak posture and ataxia [7]. Social withdrawal and loss of language become apparent at early age. Most patients progressively develop stereotypic hand wringing or washing movements. Other frequent autistic features include expressionless face, hypersensitivity to sound, lack of eye-to-eye contact and unresponsiveness to social cues [8]. This illustrates that both specific autistic and non-autistic features characterize RTT. Importantly, the RTT autistic features are also present among the general population of autistic individuals though probably in a much lower frequency.

The modest recurrence of most identified large risk variants so far precludes the inclusion of adequate carrier numbers to evaluate the specificity of the autistic subphenotype per variant. Each of these variants on its own represents only a small proportion (at most 1–2%) of the ASD population [4], [11]. Genetic disorders such as RTT that are frequently associated with ASD have associated features such as congenital malformations or somatic disorders that enhance the chance of clinical detection. Therefore, a focus on ASD subjects ascertained for particular a well defined genetic disorder enables the inclusion of larger numbers. Importantly, similar to the newly discovered genetic variants of large effects, most genetic disorders are associated with ASD only in a fraction of affected subjects, thus the defining variants in these disorders also display incomplete penetrance. This warrants a focus on subsets of individuals with a particular genetic disorder that are diagnosed with ASD which could possibly precipitate the impact of a particular variant on autistic symptomatology [1].

As a proof of concept we studied the ASD phenotype of ASD subjects with 22q11 deletion syndrome (22q11DS) and Klinefelter syndrome (KS, 47 XXY). 22q11DS and KS subjects *without* an ASD classification were excluded. 22q11DS and KS are relatively frequent disorders affecting 1–2,000–4,000 and 1–700 respectively [12]–[14]. Both are clinically defined genetic disorders like RTT and increased rates of ASD have been described in both 22q11DS and KS subjects [15]–[17]. The presence of both disorders has also previously been described among populations of subjects with ASD [18]–[20].

The structure of the ASD phenotype associated with 22q11DS and KS was compared to a large a large genetically heterogeneous sample of ASD subjects in different statistical analyses involving standard autistic measurements. The analyses aimed to assess differences in symptom homogeneity and the feasibility of differentiation of group-specific ASD-symptom profiles.

## Results

We compared the autism symptom profile of 39 subjects with 22q11DS and ASD and 14 subjects with KS and ASD to a large genetically heterogeneous sample of 372 ASD subjects (further referred to as the heterogeneous ASD sample). Autism diagnostic interview-revised (ADI-R) algorithm variable scores were entered in the analyses to evaluate differences in symptom homogeneity and the feasibility of ASD genetic subphenotype discrimination. Table 1 shows the basic characteristics of the 22q11DS-ASD, KS-ASD and heterogeneous ASD samples. Correlations in the heterogeneous ASD sample between IQ scores and the different ADI-R outcome scores were virtually negligible (−0.10<r<0.10). Therefore IQ was considered irrelevant to the outcome of the statistical comparisons with the genetic disorder ASD samples.

**Table 1:** Sample characteristics.

		Gender			
Sample	N	F	M	Age ± SD	TIQ ± SD
**22q11DS-ASD**	39	18	21	13.2±2.6	66.1±13.3
**KS-ASD**	14	0	14	13.7±3.0	81.5±13.0
**Heterogeneous ASD**	372	56	316	10.6±3.7	98.7±18.7

KS-ASD  =  Klinefelter syndrome with Autism Spectrum Disorder, 22q11DS-ASD  =  22q11 deletion syndrome with Autism Spectrum Disorder. F =  Female, M = Male, TIQ =  average total IQ score.

### Symptom homogeneity

**Figure 1:**
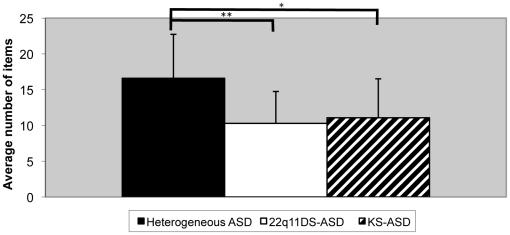
Mean number of ADI-R items reaching autistic criterion (ADI-R score  =  2). * *P*<0.002, ** *P*<0.0001, univariate analysis of variance.

Symptom homogeneity was operationalized as the (inverse of the) mean number of ADI-R algorithm symptom (called “items” in the ADI-R, see Tables S1 and S2) scores within each ASD group on which the subjects scored clearly in the autistic range, i.e. ADI-R item score  = 2. The mean number of ADI-R items on which the subjects with ASD reached the autism criterion scores differed significantly between the genetic disorder (22q11DS-ASD and KS-ASD) ASD samples and the heterogeneous ASD sample [F(2,423)  = 24.13, *P*<.0001, η_p_^2^  = .102] which indicates increased symptom homogeneity in both the genetic disorder ASD samples. Post-hoc multiple comparisons with Bonferroni correction, showed that the average number of ADI-R items that reached the ADI-R autism criterion score (eg, score  = 2) in the 22q11DS (10.28 items, SD = 4.46) and the KS (11.07 items, SD = 5.44) subjects with ASD was much lower (*P*<.00001, resp. *P*<.002) than in the heterogeneous ASD sample (16.59 items, SD = 6.14) (see Figure 1).

### Discrimination of symptom profiles

Discriminant analyses (DA) were performed to determine whether the genetic disorder ASD subsamples (22q11DS and KS with ASD) could be differentiated from the heterogeneous ASD sample on the basis of ADI-R label scores and ADI-R item scores, respectively. The success of the predictive ability of extracted ADI-R variables in the DAs is reflected in classification matrices that show the number and percentage of correctly and incorrectly identified subjects.

#### 22q11DS-ASD versus heterogeneous ASD

Discriminant analysis (DA) involving ADI-R domain subscale (called “labels” in the ADI-R, see Table S1) scores extracted labels S3, C1, R3, and R4 which resulted in a correct classification of 80% of the 22q11DS subjects and 78% of the heterogeneous ASD subjects. Box's M test for equal population covariance matrices was not significant (*P* = 0.35). Wilks' lambda  = 0.83, χ^2^ (5)  = 74.5, *P*<.0001. In the DA of the autism diagnostic interview-revised (ADI-R) algorithm items, 12 items were extracted. This resulted in a correct classification of 95% of the 22q11DS subjects and 93% of the heterogeneous ASD subjects (Table 2). Box's M test for equal population covariance matrices was not significant (P = 0.08). Wilks' lambda  = 0.58, χ^2^ (12) = 217.7, *P*<.0001. Table 3 and Table 4 state the description of the extracted labels and items respectively with the discriminant coefficients for the DA of 22q11DS+ASD versus heterogeneous ASD.

**Table 2:** Classification matrix of the discriminant analyses of 22q11DS-ASD and KS-ASD versus heterogeneous ASD through ADI-R labels and items respectively.

Sample		Number of ADI-R labels extracted	Correctly predicted % (n)	Number of ADI-R items extracted	Correctly predicted % (n)
**22q11DS-ASD vs Heterogeneous ASD**	n = 39	4	80% (31)	12	95% (37)
	n = 372		78% (290)		93% (346)
**KS-ASD vs Heterogeneous ASD**	n = 14	1	86% (12)	3	71% (10)
	n = 372		65% (242)		80% (298)

**Table 3:** Description of extracted ADI-R labels with discriminant function coefficients for the discriminant analyses of 22q11DS-ASD and KS-ASD versus heterogeneous ASD.

Sample	Label no	Label description	Function
**22q11DS-ASD**	S3	Lack of shared enjoyment	.417
	C1	Lack of, or delay in, spoken language and failure to compensate through gesture	.669
	R3	Stereotyped and repetitive motor mannerisms	.274
	R4	Preoccupations with part of objects or non-functional elements of material	.315
**KS-ASD**	S2	Relative failure to initiate or sustain conversational interchange	1.000

Domain S  =  Qualitative Abnormalities in Reciprocal Social Interaction, Domain C  =  Qualitative Abnormalities in Communication, Domain R  =  Restricted, Repetitive, and Stereotyped Patterns of Behavior.

**Table 4:** Description of extracted ADI-R items with discriminant function coefficients for the discriminant analyses of 22q11DS-ASD and KS-ASD versus heterogeneous ASD.

Sample	Item no	Item description	Domain	Function
**22q11DS-ASD**	38	Neologisms/Idiosyncratic Language	C	0,215
	43	Nodding	C	0,270
	45	Conventional/Instrumental Gestures	C	0,229
	49	Imaginative Play With Peers	S	0,192
	50	Direct Gaze	S	0,230
	51	Social Smiling	S	0,214
	52	Showing and Directing Attention	S	0,313
	57	Range of Facial Expressions Used to Communicate	S	−0,609
	58	Inappropriate Facial Expressions	S	0,221
	67	Unusual Preoccupations	R	−0,648
	68	Circumscribed Interests	R	0,352
**KS-ASD**	34	Social Verbalization/Chat	C	.215
	53	Offering to Share	S	.270
	62	Interest in Children	S	.229

Domain S  =  Qualitative Abnormalities in Reciprocal Social Interaction, Domain C  =  Qualitative Abnormalities in Communication, Domain R  =  Restricted, Repetitive, and Stereotyped Patterns of Behavior.

#### KS-ASD versus heterogeneous ASD

In the DA of KS-ASD versus heterogeneous ASD involving ADI-R labels, only label A2 was extracted which resulted in a correct classification of 86% of the KS subjects and 65% of the heterogeneous ASD subjects. Box's M test for equal population covariance matrices was not significant (*P* = 0.91). Wilks' lambda  = 0.97, χ^2^ (1)  = 10.4, *P* = .001. In the DA involving the autism ADI-R items, only 3 out of 37 items were extracted. This resulted in a correct classification of 71% of the KS subjects and 80% of the heterogeneous subjects with ASD (Table 2). Box's M test was not significant (*P* = 0.63). Wilks' lambda  = 0.91, χ^2^ (3) = 34.50, *P*<.001. Table 3 and Table 4 state the description of the extracted labels and items respectively with the discriminant coefficients for the DA of KS-ASD versus heterogeneous ASD.

**Figure 2:**
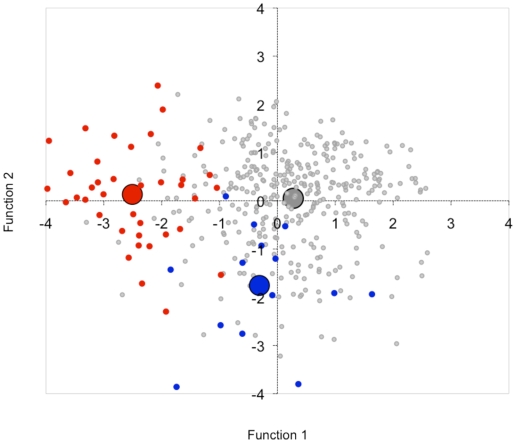
Plot of individual subject canonical function coefficients of the 3-group discriminant analysis for heterogeneous ASD (grey dots) versus 22q11DS-ASD (red dots) versus KS-ASD (blue dots), the larger dots represent the group centroids. 22q11DS-ASD is predominantly discriminated from heterogeneous ASD by function 1, KS-ASD from heterogeneous ASD by function 2.

A three-group DA (ie 22q11-ASD vs. KS-ASD vs. heterogeneous ASD) involving ADI-R items resulted in a correct classification of 92.3% of the 22q11DS-ASD subjects, 78.6% of the KS-ASD subjects, and 76.4% of the heterogeneous ASD subjects (see Table S3) on the basis of 12 extracted items. Box's M test was not significant (*P* = 0.25). Wilks' lambda for function 1 = 0.55, χ^2^ (24) = 250.86, *P*<.0001, and for function 2 Wilks' lambda  = 0.90, χ^2^ (11) = 42.04, *P*<.0001. The 3-group percentages were similar to the results of the individual 22q11DS-ASD and KS-ASD to heterogeneous ASD ADI-R item comparisons. Figure 2 is the plot of the individual discriminant coefficients of the 3-group discriminant analysis. It illustrates that 22q11DS-ASD is predominantly discriminated from heterogeneous ASD by function 2, KS-ASD from heterogeneous ASD by function 1. Table S4 states the description of the extracted items with the discriminant coefficients for the 3-group DA. No additional items were extracted for the three-group comparison than had been extracted in both separate 22q11DS-ASD and KS-ASD versus heterogeneous ASD comparisons. In addition, 4 items that were extracted in the 2-group comparisons were not extracted for the 3-group comparisons (items 38, 49 and 51 out of the 22q11DS-ASD versus Heterogeneous ASD sample and item 62 out of the KS-ASD versus heterogeneous ASD comparisons).

Table S5 provides an overview of all the extracted ADI-R in the different DAs. Table S6 contains a verifying calculation regarding the stability of the DA results.

## Discussion

Our results indicate that the clinical heterogeneity of ASDs might be reduced when subgroups based on a specific genotype are extracted from the overall genetically heterogeneous ASD population. A substantially lower autistic symptom variance was shown in both the 22q11DS-ASD and KS-ASD samples in comparison to the heterogeneous ASD sample. Further analysis revealed that the symptom profiles of the two studied genetic ASD disorders could be robustly discriminated from the heterogeneous ASD profiles through a limited number of autistic subscales and symptoms. In our opinion, these results support the notion of the existence of genetic subphenotypes within the ASD population. The lack of overlap in the discriminating ADI-R labels and items between KS-ASD and 22q11DS-ASD could indicate that both syndromes represent ASD profiles that cluster around different specific points in the total ASD diagnostic space, which was also suggested by the results of the three-group analysis. It should be emphasized that genetic disorder ASD subphenotypes are expected to overlap with profiles present among heterogeneous samples, as all are diagnosed according to the same DSM-IV-TR/ADI-R criteria. The premise of an overlap of ASD subphenotypes between carriers and non-carriers of large impact variants raises interesting questions. It could be speculated that autistic symptom profiles out of the heterogeneous ASD population that overlap with symptom profiles related to specific genotypes can possibly point to convergent etiologies.

Several limitations should be addressed. The data were gathered from different studies, however, all studies were performed by the authors of this paper, and the diagnostic instruments were identical between the studies. The 22q11DS-ASD and KS-ASD subjects were not selected form an original ASD sample, but derived from psychiatric surveys among children with 22q11DS and KS samples. It would have been preferable if all subjects had been recruited in the same way. However, this would require unfeasibly large ASD samples to extract a sufficient number of 22q11-DS and KS-ASD cases. Although the ASD subjects with 22q11DS and KS and the heterogeneous ASD subjects fulfilled the same DSM-IV-TR clinical and ADI-R criteria, the average clinical threshold for suspecting ASD could have been different in the heterogeneous sample. Therefore, ascertainment bias cannot be ruled out. However, we did not aim to validate the association of 22q11DS and KS with ASD but rather investigated whether specific genotypes can confer specific autistic symptom profiles.

We do realize that reliability for the ADI-R is typically at the level of overall diagnosis, or subdomain area and that the ADI-R was not originally designed as a dimensional measure. Nonetheless we found the largest contrast between the different groups at the level of individual symptom items, while the DA involving ADI-R label subscales also delivered better results than expected by chance.

The sample sizes in this study preclude conclusions towards the nature of the discrimination symptoms. Similar assessments in other larger ASD cohorts are required to prove whether the current approach is feasible for “reversed phenotype” efforts. Additional measures such as the Social Responsiveness Scale or the Autism Diagnostic Observation Schedule (ADOS) can possibly aid to enhance specificity of phenotype descriptions [21], [22].

The current findings could suggest that the KS and 22q11DS genotypes do not seem to merely augment heterogenic and complex genetic susceptibility, i.e. lower the ASD threshold in an aspecific way. Rather, our results could suggest that the strong influence of a specific genetic variant leads to an ASD subphenotype that is relatively specific with an increased within-group symptom homogeneity in comparison to the heterogeneous ASD population. Based upon these observations we hypothesize that the increased symptom homogeneity is mainly driven by the effect of one (or limited) genetic pathway(s). In contrast, the phenotype observed in the general ASD population is most likely mediated by the interplay of various combinations of all culprit causative genetic pathways, and therefore associated with larger ASD symptom heterogeneity. This consideration was emphasized in recent overview of advancements in genetic studies of complex traits: “For a substantial number of common diseases the newly identified pathways suggest that molecular subphenotypes may exist; that is, although a number of different pathways might potentially be involved in the development of a particular disease when all cases are considered, in any individual with the disease only one or a subset of these pathways might be involved” [23]. Other ASD subphenotypes related to newly identified genetic variants (e.g. 1q21 duplication or deletion, the 22q13.3 deletion and the duplication of the 15q11–13 region) may be identified when properly studied. This could ultimately lead to a dissection of the ASD phenotype into a proportion of “genetic subtypes”, and a remaining group of ASD patients in whom the ASD phenotype is the resultant of a more complex interaction between common genetic variants and environmental factors.

In conclusion, the current findings support the possibility that reduced genetic heterogeneity can be associated with reduced ASD symptom heterogeneity. The method of the current study using symptom variance and discrimination analysis involving the widely used ADI-R offers a relatively straightforward possibility for assessing genotype-ASD relationships. The assessments aim to initiate further reverse phenotype strategies, especially given the accumulating evidence for the existence of genetic variants of large effect in a substantial proportion of the ASD population.

## Methods

### Participants

The Dutch Central Committee on Research Involving Human Subjects had approved the research protocol. Patient associations and centers for clinical genetics, and pediatrics were involved in recruiting the children for the original psychiatric surveys out of which the subjects for the present study were selected (see below). A newsletter presented on the web or in writing, had informed parents and children of the aim and methods of the study. Parents and children of had to apply actively for participation in the study by contacting the research team. Subsequently they were sent written information about the selection criteria and the implications of participation in the study. They were invited for assessment if they met the inclusion criteria. Written informed consent was obtained from participants (if older than 12 years of age) and their parents or guardians according to the declaration of Helsinki.

22q11DS or KS subjects were selected for this study if they had been diagnosed with an ASD via previous surveys on general psychopathology on children with 22q11DS (n = 90) and Klinefelter Syndrome (KS) sample (n = 51), all 47, XXY none higher aneuploidies, no mosaics). (See [16], [17] for more extensive details on recruitment and further characteristics of the patient samples). This survey had resulted in an ASD classification in 14 of the 51 KS boys (14/51 = 27%) and 39 of the 90 22q11DS children (39/90 = 43%). The classification of an autism spectrum disorder (ASD) was made on the basis of DSM-IV-TR standardized interviews and the Autism Diagnostic Interview (ADI-R) [24]. Videotapes of all subjects and the DSM-IV-TR/ADI-R outcomes were discussed in a consensus meeting headed by the head of the department. The consensus meeting served to control for procedural mistakes and to verify whether the classifications through the DSM-IV-TR and ADI-R interviews were in agreement with the clinical judgment. All 22q11DS and KS subjects with an ASD met ADI-R thresholds and DSM-IV-TR criteria.

A genetically heterogeneous ASD sample was recruited as part of a genetic study of autism and from a clinical sample of patients referred to the department of child and adolescent psychiatry for diagnostic reasons. Thus, these subjects were unascertained for their genotype and should therefore represent a reference sample of maximal genetic heterogeneity. Inclusion criteria were: age four years or older, no severe medical or neurological illness, IQ>40. The final sample consisted of 372 verbal subjects. Study participants ranged in age from 4 to 20 years. Similar to the ASD cases obtained from the 22q11 and XXYDS cohorts, all subjects out of the heterogeneous ASD sample had been evaluated in consensus meetings to confirm ASD diagnosis through the interviews and all subjects met ADI-R thresholds.

IQ had been assessed by means of the Dutch versions of the Wechsler scales (WPSSI WISC III and WAIS) [25]–[27] in the KS and 22q11DS sample and in a significant part of the ASD heterogeneous group (65%). IQ scores of the heterogeneous ASD sample that could not be assessed with the Wechsler scales have been assessed with the RAVEN Progressive Matrices [28] the Mullen Scales of Early Learning [29] or the Snijders-Oomen non-verbal intelligence test-Revised [30]. No difference in intelligence level between those with and those without ASDs had been found in both the 22q11DS and KS samples in the original psychiatric surveys (see [16], [17]).

### Measurements

ADI-R subscale and symptom variables were entered in the statistical analyses for phenotype comparisons. The ADI–R is an established ‘gold standard’ in diagnostic/phenotypic evaluations of autism. It is an extensive clinical interview administered to the parents. The interview focuses on the three core or so called “content” domains of autism (i.e. qualitative abnormalities in social interaction (S), qualitative abnormalities in communication (C) and stereotyped and repetitive behaviors (R) [24]. ADI-R items are coded for these domains and also for an “age of onset” domain. A classification of an autism spectrum disorder is applied when scores in all domains are met or when scores are met in two core domains and meet criteria on the “age of onset” domain, but are one point away from meeting autism criteria in the remaining core domain. Reliability of the ADI-R in a population with mild to moderate mental retardation has been established [31].

The ADI-R may also be used to assess profiles of autistic symptomatology [32], [33]. The ADI–R algorithm is composed of 37 symptom “items”. These items were originally selected as a minimum for optimal ASD classification [24]. ADI-R labels consist of 2 to 5 items and are directly related to the DSM-IV-TR criteria of an Autistic Disorder. As a result, 12 ADI-R labels are used. Each ADI-R domain consists of 4 labels, eg S1-4, C1-4 and R1-4. Items are coded as 0 (ASD behavioural symptom specified not present), 1 (specified behaviour not sufficient to code “2”) or 2 (specified ASD symptom present). Maximum label scores thus range from 4–10. An overview of the description of the ADI-R items, labels and the ADI-R domains of the algorithm is provided in Tables S1 and S2.

### Statistical analyses

Symptom homogeneity was operationalized as the (inverse of the) mean number of ADI-R algorithm items within each ASD group on which the subjects scored clearly in the autistic range, i.e. ADI-R item score  = 2. Thus, per subject, the number of items with score  = 2 were counted. A lower number of ADI-R items that reached the autism criterion can be considered indicative of a relative reduction in symptom heterogeneity. Differences between groups in number of ADI-R items on which the autism criterion was reached were analyzed by means of a univariate analysis of variance, with post hoc Bonferroni multiple comparisons.

Discriminant analyses (DA) were performed to determine whether the genetic disorder ASD subsamples could be differentiated from the heterogeneous ASD sample on the basis of ADI-R variables. The analyses addressed the question to what extent ADI-R label and/or ADI-R symptom item profiles could successfully discriminate 22q11DS or KS ASD profiles from the heterogeneous ASD sample profile (i.e., 22q11DS+ASD vs. heterogeneous ASD, and KS+ASD vs. heterogeneous ASD). In addition, three group DAs were performed to explore the separation of the three groups by means of 2 discriminant functions.

For all discriminant analyses, stepwise Wilks' lambda was used, with probability of F for entry and removal of variables set at 0.5 and 0.10 respectively. For classification the within-group covariance matrices were used and prior probability was set to equal for all groups.

## Supporting Information

Table S1 ADI-R algorithm items sorted by labels and domains.
(0.07 MB DOC)

Table S2 ADI-R algorithm items sorted by number.
(0.05 MB DOC)

Table S3 Classification matrix of the 3-group discriminant analysis of heterogeneous ASD versus KS-ASD versus 22q11DS-ASD.
(0.04 MB DOCX)

Table S4 Description and discriminant function coefficients of ADI-R items extracted in the 3-group discriminant analysis of heterogeneous ASD versus KS-ASD versus 22q11DS-ASD.
(0.04 MB DOC)

Table S5 Overview of extracted ADI-R items with subsequent labels in the different discriminant analyses. DA1  = 22q11DS-ASD versus heterogeneous ASD. DA2  =  KS-ASD versus heterogeneous ASD. D3  = 3-group group comparison of 22q11DS-ASD versus KS-ASD versus heterogeneous ASD.
(0.05 MB DOC)

Table S6 Results for stability calculations of DAs. ADI-R items extracted in the 4 additional DAs, sorted by correspondence/item number. To verify whether the stepwise analysis of the three groups DA did provide stable solution, the stepwise DA to separate the three groups has been repeated another four times, with half of the sample of subjects with heterogeneous ASD. Thereto the sample was divided in four quarters, called Q1 to Q4. The DA has been performed with inclusion of the heterogeneous subsamples Q1+Q2, Q3+Q4, Q1+Q3, and Q2+Q4. The ‘solutions’ are compared with each other and with the result of the DA presented in the paper that included the total sample of subjects with heterogeneous ASD. The comparison is focused on the number and type of items that are extracted. Table S6 shows that the solutions are highly similar. The number of items extracted varies between 10 and 15. There are 8 items that appear in each DA, there is 1 items that appears in 3 DAs, there are 7 items that appear in 2 DAs, there is 1 item appearing in only one DA. Comparing the four solutions with the results of the original DA presented in the paper shows that all of the 12 items extracted in this DA, all show up in one or more one of the other DAs. Eight items of the original DA show up in all other analyses, the other four items of the original DA appear in at least two of the other DAs.
(0.06 MB DOC)
