# Supplementary material for: Dissecting the Clinical Heterogeneity of Autism Spectrum Disorders through Defined Genotypes
Source: PLoS One. 2010 May 28;5(5):e10887. doi: 10.1371/journal.pone.0010887 (PMC2878316; doi:10.1371/journal.pone.0010887)
Supplement: Table S1 — ADI-R algorithm items sorted by labels and domains. (0.07 MB DOC) [file pone.0010887.s001.doc]

|  | **S: Qualitative Abnormalities in Reciprocal Social Interaction** |
| --- | --- |
|  | **S1: Failure to use nonverbal behaviors to regulate social interaction** |
| 50 | Direct Gaze |
| 51 | Social Smiling |
| 57 | Range of Facial Expressions Used to Communicate |
|  | **S2: Failure to develop peer relationships** |
| 49 | Imaginative Play With Peers |
| 62 | Interest in Children |
| 63 | Response to Approaches of Other Children |
| 64 | Group Play with Peers (age < 10.0) |
| 65 | Friendships (age > 10.0) |
|  | **S3: Lack of shared enjoyment** |
| 52 | Showing and Directing Attention |
| 53 | Offering to Share |
| 54 | Seeking to Share Enjoyment With Others |
|  | **S4: Lack of socioemotional reciprocity** |
| 31 | Use of Other’s Body to Communicate |
| 55 | Offering Comfort |
| 56 | Quality of Social Overtures |
| 58 | Inappropriate Facial Expressions |
| 59 | Appropriateness of Social Responses |
|  | **C: Qualitative Abnormalities in Communication** |
|  | **C1: Lack of, or delay in, spoken language and failure to compensate through gesture** |
| 42 | Pointing to Express Interest |
| 43 | Nodding |
| 44 | Head Shaking |
| 45 | Conventional/Instrumental Gestures |
|  | **C4: Lack of varied spontaneous make-believe or social imitative play** |
| 47 | Spontaneous Imitation of Actions |
| 48 | Imaginative Play |
| 61 | Imitative Social Play |
|  | **C2(V): Relative failure to initiate or sustain conversational interchange** |
| 34 | Social Verbalization/Chat |
| 35 | Reciprocal Conversation |
|  | **C3(V): Stereotyped, repetitive or idiosyncratic speech** |
| 33 | Stereotyped Utterances and Delayed Echolalia |
| 36 | Inappropriate Questions or Statements |
| 37 | Pronominal Reversal |
| 38 | Neologisms/Idiosyncratic Language |
|  | **R: Restricted, Repetitive, and Stereotyped Patterns of Behavior** |
|  | **R1: Encompassing preoccupation or circumscribed pattern of interest** |
| 67 | Unusual Preoccupations |
| 68 | Circumscribed Interests |
|  | **R2: Apparently compulsive adherence to nonfunctional routines or rituals** |
| 39 | Verbal Rituals |
| 70 | Compulsions/Rituals |
|  | **R3: Stereotyped and repetitive motor mannerisms** |
| 77 | Hand and Finger Mannerisms (score highest of 77/78) |
| 78 | Other Complex Mannerisms or Stereotyped Body Movements |
|  | **R4: Preoccupations with part of objects or non-functional elements of material** |
| 69 | Repetitive Use of Objects or Interest in Parts of Objects |
| 71 | Unusual Sensory Interests (score highest of 69/71) |

**Table S1:** ADI-R algorithm items sorted by labels and domains.
