# Supplementary material for: Dissecting the Clinical Heterogeneity of Autism Spectrum Disorders through Defined Genotypes
Source: PLoS One. 2010 May 28;5(5):e10887. doi: 10.1371/journal.pone.0010887 (PMC2878316; doi:10.1371/journal.pone.0010887)
Supplement: Table S2 — ADI-R algorithm items sorted by number. (0.05 MB DOC) [file pone.0010887.s002.doc]

| **Item no** | **Item description** | **Domain label** |
| --- | --- | --- |
| 31 | Use of Other’s Body to Communicate | S4 |
| 33 | Stereotyped Utterances and Delayed Echolalia | R3 |
| 34 | Social Verbalization/Chat | B2 |
| 35 | Reciprocal Conversation | B2 |
| 36 | Inappropriate Questions or Statements | B3 |
| 37 | Pronominal Reversal | B3 |
| 38 | Neologisms/Idiosyncratic Language | B3 |
| 39 | Verbal Rituals | R2 |
| 42 | Pointing to Express Interest | B1 |
| 43 | Nodding | B1 |
| 44 | Head Shaking | B1 |
| 45 | Conventional/Instrumental Gestures | B1 |
| 47 | Spontaneous Imitation of Actions | B4 |
| 48 | Imaginative Play | B4 |
| 49 | Imaginative Play With Peers | S2 |
| 50 | Direct Gaze | S1 |
| 51 | Social Smiling | S1 |
| 52 | Showing and Directing Attention | S3 |
| 53 | Offering to Share | S3 |
| 54 | Seeking to Share Enjoyment With Others | S3 |
| 55 | Offering Comfort | S4 |
| 56 | Quality of Social Overtures | S4 |
| 57 | Range of Facial Expressions Used to Communicate | S1 |
| 58 | Inappropriate Facial Expressions | S4 |
| 59 | Appropriateness of Social Responses | S4 |
| 61 | Imitative Social Play | B4 |
| 62 | Interest in Children | S2 |
| 63 | Response to Approaches of Other Children | S2 |
| 64 | Group Play with Peers (age < 10.0) | S2 |
| 65 | Friendships (age > 10.0) | S2 |
| 67 | Unusual Preoccupations | R1 |
| 68 | Circumscribed Interests | R1 |
| 69 | Repetitive Use of Objects or Interest in Parts of Objects | R4 |
| 70 | Compulsions/Rituals | R2 |
| 71 | Unusual Sensory Interests (score highest of 69/71) | R4 |
| 77 | Hand and Finger Mannerisms (score highest of 77/78) | R3 |
| 78 | Other Complex Mannerisms or Stereotyped Body Movements | R3 |

**Table S2**: ADI-R algorithm items sorted by number.
