# Supplementary material for: Dissecting the Clinical Heterogeneity of Autism Spectrum Disorders through Defined Genotypes
Source: PLoS One. 2010 May 28;5(5):e10887. doi: 10.1371/journal.pone.0010887 (PMC2878316; doi:10.1371/journal.pone.0010887)
Supplement: Table S3 — Classification matrix of the 3-group discriminant analysis of heterogeneous ASD versus KS-ASD versus 22q11DS-ASD. (0.04 MB DOCX) [file pone.0010887.s003.docx]

|  | **Correctly predicted % (n)** | **Incorrectly predicted % (n)** | | |
| --- | --- | --- | --- | --- |
| Diagnostic groups |  | Heterogeneous | 22q11DS | KS |
| Heterogeneous ASD | 76.4 % (285) |  | 6.4 % (24) | 17.2 % (64) |
| 22q11DS-ASD | 92.3 % (36) | 2.6 % (1) |  | 5.1 % (2) |
| KS-ASD | 78.6 % (11) | 21.4 % (3) |  | 0.0 % (0) |

**Table S3:** Classification matrix of the 3-group discriminant analysis of heterogeneous ASD versus

KS-ASD versus 22q11DS-ASD.
