# Supplementary material for: Dissecting the Clinical Heterogeneity of Autism Spectrum Disorders through Defined Genotypes
Source: PLoS One. 2010 May 28;5(5):e10887. doi: 10.1371/journal.pone.0010887 (PMC2878316; doi:10.1371/journal.pone.0010887)
Supplement: Table S4 — Description and discriminant function coefficients of ADI-R items extracted in the 3-group discriminant analysis of heterogeneous ASD versus KS-ASD versus 22q11DS-ASD. (0.04 MB DOC) [file pone.0010887.s004.doc]

| **Item no** | **Item description** | **Domain** | **Function** | |
| --- | --- | --- | --- | --- |
|  |  |  | 1 | 2 |
| 34 | Social Verbalization/Chat | C | .130 | .383 |
| 43 | Nodding | C | .260 | -.404 |
| 45 | Conventional/Instrumental Gestures | C | .207 | .233 |
| 50 | Direct Gaze | S | .269 | -.131 |
| 52 | Showing and Directing Attention | S | .274 | -.410 |
| 53 | Offering to Share | S | .092 | .677 |
| 57 | Range of Facial Expressions Used to Communicate | S | -.561 | .071 |
| 58 | Inappropriate Facial Expressions | S | .266 | .033 |
| 62 | Interest in Children | S | .141 | .417 |
| 67 | Unusual Preoccupations | R | -.636 | .154 |
| 68 | Circumscribed Interests | R | .387 | .113 |
| 6971 | Repetitive Use of Objects or Interest in Parts of Objects, or  Unusual Sensory Interests | R | .340 | -.079 |

**Table S4:** Description and discriminant function coefficients of ADI-R items extracted in the 3-group discriminant analysis of heterogeneous ASD versus KS-ASD versus 22q11DS-ASD.
