# Supplementary material for: Dissecting the Clinical Heterogeneity of Autism Spectrum Disorders through Defined Genotypes
Source: PLoS One. 2010 May 28;5(5):e10887. doi: 10.1371/journal.pone.0010887 (PMC2878316; doi:10.1371/journal.pone.0010887)
Supplement: Table S5 — Overview of extracted ADI-R items with subsequent labels in the different discriminant analyses. DA1 = 22q11DS-ASD versus heterogeneous ASD. DA2 = KS-ASD versus heterogeneous ASD. D3 = 3-group group comparison of 22q11DS-ASD versus KS-ASD versus heterogeneous ASD. (0.05 MB DOC) [file pone.0010887.s005.doc]

| **Item no** | **Domain** | **Item description** | **DA1** | **DA2** | **DA3** |
| --- | --- | --- | --- | --- | --- |
| 34 | C | Social Verbalization/Chat |  | x | x |
| 38 | C | Neologisms/Idiosyncratic Language | x |  |  |
| 43 | C | Nodding | x |  | x |
| 45 | C | Conventional/Instrumental Gestures | x |  | x |
| 49 | S | Imaginative Play | x |  |  |
| 50 | S | Direct Gaze | x |  | x |
| 51 | S | Social smiling | x |  |  |
| 52 | S | Showing and Directing Attention | x |  | x |
| 53 | S | Offering to Share |  | x | x |
| 57 | S | Range of Facial Expressions Used to Communicate | x |  | x |
| 58 | S | Inappropriate Facial Expressions | x |  | x |
| 62 | S | Interest in Children |  | x | x |
| 67 | R | Unusual Preoccupations | x |  | x |
| 68 | R | Circumscribed Interests | x |  | x |
| 6971 | R | Repetitive Use of Objects or Interest in Parts of  Objects / Unusual Sensory Interests | x |  | x |

**Table S5:** Overview of extracted ADI-R items with subsequent labels in the different

discriminant analyses. DA1= 22q11DS-ASD versus heterogeneous ASD. DA2= KS-ASD

versus heterogeneous ASD. D3= 3-group group comparison of 22q11DS-ASD versus

KS-ASD versus heterogeneous ASD.
