# Supplementary material for: Dissecting the Clinical Heterogeneity of Autism Spectrum Disorders through Defined Genotypes
Source: PLoS One. 2010 May 28;5(5):e10887. doi: 10.1371/journal.pone.0010887 (PMC2878316; doi:10.1371/journal.pone.0010887)
Supplement: Table S6 — Results for stability calculations of DAs. ADI-R items extracted in the 4 additional DAs, sorted by correspondence/item number. To verify whether the stepwise analysis of the three groups DA did provide stable solution, the stepwise DA to separate the three groups has been repeated another four times, with half of the sample of subjects with heterogeneous ASD. Thereto the sample was divided in four quarters, called Q1 to Q4. The DA has been performed with inclusion of the heterogeneous subsamples Q1+Q2, Q3+Q4, Q1+Q3, and Q2+Q4. The ‘solutions’ are compared with each other and with the result of the DA presented in the paper that included the total sample of subjects with heterogeneous ASD. The comparison is focused on the number and type of items that are extracted. Table S6 shows that the solutions are highly similar. The number of items extracted varies between 10 and 15. There are 8 items that appear in each DA, there is 1 items that appears in 3 DAs, there are 7 items that appear in 2 DAs, there is 1 item appearing in only one DA. Comparing the four solutions with the results of the original DA presented in the paper shows that all of the 12 items extracted in this DA, all show up in one or more one of the other DAs. Eight items of the original DA show up in all other analyses, the other four items of the original DA appear in at least two of the other DAs. (0.06 MB DOC) [file pone.0010887.s006.doc]

|  |  | Samples | | | | |
| --- | --- | --- | --- | --- | --- | --- |
| **Item** | **Item description** | **Q1+Q2** | **Q3+Q4** | **Q1+Q3** | **Q2+Q4** | **ALL** |
| 43 | Nodding | x | x | x | x | x |
| 50 | Direct Gaze | x | x | x | x | x |
| 53 | Offering to Share | x | x | x | x | x |
| 57 | Range of Facial Expressions Used to Communicate | x | x | x | x | x |
| 58 | Inappropriate Facial Expressions | x | x | x | x | x |
| 67 | Unusual Preoccupations | x | x | x | x | x |
| 68 | Circumscribed Interests | x | x | x | x | x |
| 6971 | Repetitive Use of Objects or Interest in Parts of Objects/ Unusual Sensory Interests | x | x | x | x | x |
| 52 | Showing and Directing Attention | x |  | x | x | x |
| 45 | Conventional/Instrumental Gestures | x |  |  | x | x |
| 49 | Imaginative Play With Peers | x |  | x |  |  |
| 51 | Social Smiling |  | x | x |  |  |
| 62 | Interest in Children |  | x | x |  | x |
| 70 | Compulsions/Rituals | x |  |  | x |  |
| 7778 | Hand and Finger Mannerisms / Other Complex Mannerisms or Stereotyped Body Movements | x |  |  | x |  |
| 34 | Social Verbalization/Chat | x |  |  | x | x |
| 38 | Neologisms/Idiosyncratic Language | x |  |  |  |  |
|  |  | N = 15 | N = 10 | N = 12 | N = 13 | N = 12 |

**Table S6:** Results for stability calculations of DAs. ADI-R items extracted in the 4 additional DAs, sorted by correspondence/item number. To verify whether the stepwise analysis of the three groups DA did provide stable solution, the stepwise DA to separate the three groups has been repeated another four times, with half of the sample of subjects with heterogeneous ASD. Thereto the sample was divided in four quarters, called Q1 to Q4. The DA has been performed with inclusion of the heterogeneous subsamples Q1+Q2, Q3+Q4, Q1+Q3, and Q2+Q4. The ‘solutions’ are compared with each other and with the result of the DA presented in the paper that included the total sample of subjects with heterogeneous ASD. The comparison is focused on the number and type of items that are extracted. Table S6 shows that the solutions are highly similar. The number of items extracted varies between 10 and 15. There are 8 items that appear in each DA, there is 1 items that appears in 3 DAs, there are 7 items that appear in 2 DAs, there is 1 items appearing in only one DA. Comparing the four solutions with the results of the original DA presented in the paper shows that all of the 12 items extracted in this DA, all show up in one or more one of the other DAs. Eight items of the original DA show up in all other analyses, the other four items of the original DA appear in at least two of the other DAs.
